# Biochar, Bentonite and Zeolite Supplemented Feeding of Layer Chickens Alters Intestinal Microbiota and Reduces *Campylobacter* Load

**DOI:** 10.1371/journal.pone.0154061

**Published:** 2016-04-26

**Authors:** Tanka P. Prasai, Kerry B. Walsh, Surya P. Bhattarai, David J. Midmore, Thi T. H. Van, Robert J. Moore, Dragana Stanley

**Affiliations:** 1 Central Queensland University, Institute for Future Farming Systems, Rockhampton, Queensland, Australia; 2 RMIT University, School of Applied Sciences and Health Innovations Research Institute (HIRI), Bundoora, Victoria, Australia; 3 Department of Microbiology, Monash University, Clayton, Victoria, Australia; Max Rubner-Institut, GERMANY

## Abstract

A range of feed supplements, including antibiotics, have been commonly used in poultry production to improve health and productivity. Alternative methods are needed to suppress pathogen loads and maintain productivity. As an alternative to antibiotics use, we investigated the ability of biochar, bentonite and zeolite as separate 4% feed additives, to selectively remove pathogens without reducing microbial richness and diversity in the gut. Neither biochar, bentonite nor zeolite made any significant alterations to the overall richness and diversity of intestinal bacterial community. However, reduction of some bacterial species, including some potential pathogens was detected. The microbiota of bentonite fed animals were lacking all members of the order Campylobacterales. Specifically, the following operational taxonomic units (OTUs) were absent: an OTU 100% identical to *Campylobacter jejuni*; an OTU 99% identical to *Helicobacter pullorum*; multiple *Gallibacterium anatis* (>97%) related OTUs; *Bacteroides dorei* (99%) and *Clostridium aldenense* (95%) related OTUs. Biochar and zeolite treatments had similar but milder effects compared to bentonite. Zeolite amended feed was also associated with significant reduction in the phylum Proteobacteria. All three additives showed potential for the control of major poultry zoonotic pathogens.

## Introduction

The use of antibiotic growth promoters as feed additives to suppress the pathogenic bacteria in the gut has been common in commercial poultry production, however it is banned in Europe [1] because of concerns for the consequences it could have on human health in terms of the selection of antibiotic resistant microbiota and for the presence of residual antibiotics in poultry products [2]. Alternatives to antibiotic growth promoters are required in order to maintain bird health and deliver the productivity improvements that were sometimes associated with their use.

Laying hens are in great need of antibiotic-free pathogen control given antibiotics can not be used due to residue carry over to eggs. For example, in Queensland, Spotty Liver is emerging as a disease of concern. This disease is caused by *Campylobacter* species [3] and is currently controlled by antibiotics. Layers colonisation with human pathogens such as *Salmonella* and C*ampylobacter* is an important issue that the industry is grappling with, and for which new solutions are required. Additionally antibiotic-free pathogen control is needed in organic poultry production. There are many alternative products under investigation. Among them, biochar, bentonite and zeolite are interesting candidates for selective pathogen control as there is mounting evidence that they are safe and beneficial products [4–6]. Properties of these three natural products are outlined below.

Biochar is a carbon rich product produced from the incomplete combustion of biomass in the absence of oxygen through a process termed pyrolysis [7]. Biochar, and in particular biochar bokashi, is used as a feed supplement in Japan and China, with claims for improved digestion and feed conversion ratio [4]. More complete biochar pyrolysis results in production of charcoal. Diet supplementation with charcoal has also been reported to result in increased live weight gain and higher feed conversion ratio (FCR) for commercial meat chickens and ducks [8, 9]. Several mechanisms have been suggested for the benefit of biochar or charcoal in animal diets, including toxin binding, improved digestion and retention of nitrogen. A possible mechanism for the improved FCR associated with biochar supplements could lie with a change in gastro intestinal tract (GIT) microbiota.

Bentonite is a clay mineral with strong colloidal properties and the ability to rapidly absorb many times its volume of water. Clays are often incorporated in animal diets as a stabilizer, lubricant or agglomerant to improve feed manufacture [10]. Nutrient digestibility and enzymatic activity of gastrointestinal secretions has been improved by addition of clay to broilers and pig feedstuffs [11–14]. Bentonite has been used effectively as a feed additive in poultry rations, with the swelling of bentonite causing a reduction in the rate of feed transit through the digestive tract, permitting time for more effective utilisation [15]. Addition of sodium bentonite was effective in ameliorating the negative effect of aflatoxins in poultry diet [16]. The toxin is prevented from being absorbed by the digestive tract and the bound aflatoxin is then excreted [6]. The supplementation of poultry rations with a Cu-montmorillonite clay has been reported to result in reduced total viable counts of *Escherichia coli* and *Clostridium* in the small intestine and caecum of chicks [17].

Clinoptilolite is a common form of natural zeolite. Zeolites are crystalline, hydrated aluminosilicates of alkali and alkaline earth cations. Zeolites have cation exchange properties and are capable of trapping molecules within their pores [18]. For example, the porosity, particle and crystal size of the zeolitic material and its degree of aggregation determine the rate of access of ingesta fluids during passage through the GIT [5]. Average daily live body weight gain and feed conversions in broilers have been improved with dietary inclusion of zeolites [5, 19]. Zeolite feed amendment has also been reported to increase egg production [20] and have positive effects on egg weight and internal egg quality [5, 20]. Papaioannou *et al*. [21] reported zeolite feed amendment to be associated with a reduction in the rate of passage of feed through the digestive system, and an associated reduction in feed intake [22] resulting in better FCR. However, factors including the type of zeolite, its purity, physiochemical properties, and the supplementation level used in the diets may impact the performance effect.

Chemically modified natural zeolites have been associated with bactericidal effects on pathogenic organisms in the guts of birds. A reduction in mortality of broiler chickens and reduced viable counts of *Salmonella enteritidis* and *Escherichia coli* in the proximal and distal gut were associated with inclusion of zeolite in feed, [23]. Zeolite can be modified chemically with organic cations resulting in increased hydrophobicity of the mineral surface, increasing its adsorptive capacity to certain molecules, and resulting in increased bactericidal effects against *Escherichia coli* and its toxins [24, 25].

The mechanisms of action of biochar, bentonite and zeolite are likely to be multifactorial. In this study we investigate their effects on the gut microbiota of birds fed with these natural products. As noted above, some studies have reported changes in the carriage of a few different bacterial species in the face of these additives based on culturing of selected pathogens. Our goal was to undertake a more comprehensive analysis of the potential suppression of pathogenic microbiota induced by the additives. High throughput DNA sequencing using the 16S ribosomal RNA gene as a phylogenetic marker is a culture free method that allows analysis of all of the complex bacterial populations that make up the gut microbiota rather than only few capable of growing on selected microbiological media. The use of these technologies over the last decade has revealed the high complexity of microbiotas from many different ecological niches including the gastrointestinal tracts (GIT) of animals and humans. The evolving understanding of the many roles and biological functions that are affected by the GIT microbiota has led to the consideration of the GIT microbiota as the largest organ of the body, contributing 10 times more cells and 100 times more gene products to the host then host’s own cells. Research on chickens has shown that some commercial practices, such as the addition of antibiotics that reduce microbial diversity in the gut [26], and sanitary hatching practices that remove the influence of maternal microbiota from the hatchlings [27], lead to the formation of distorted microbial communities that may be poorly adapted to digest complex avian diets. To achieve the goal of a healthy and productive chicken GIT, free of major zoonotic and chicken pathogens, without disturbing the natural beneficial intestinal bacteria, alternative treatments and management practices are needed. These must retain the richness and diversity of the chicken GIT microbiota while selectively reducing pathogens that are potentially deleterious to the chicken or consumers of chicken products.

## Materials and Methods

### Animal trial and sample collection

All procedures involving animals were approved by the Animal Ethics Committee of the Central Queensland University (Approval number A 12/06-283). The trial was conducted in a screened shed environment with temperature variation from 22.5 to 36.5°C on the Central Queensland University Rockhampton campus, from May 2014 to December 2014. Eighty Bond Brown Layer (BBL) 17 week old pullets were obtained from Bond Enterprises P/L, Grantham, Qld, Australia. This poultry breed is a brown egg commercial layer developed by Bond Enterprises, marketed as possessing the traits of 95% peak egg production and a feed conversion ratio (FCR) of 2.1–2.3 kg feed/kg of eggs.

The pullets were allowed one week to adjust to the new environment before introducing new diet treatments. The regular commercial layer ration (Blue Ribbon Stocks Feed, Rockhampton) consisted of 90.35% dry matter, metabolisable energy 11.75 MJ/kg, crude protein 17.5% and calcium 4.2% by dry weight, supplied as a crumbed mix. A professional animal nutritionist (www.wadeagriculture.com.au/) undertook the feed formulation to maintain energy value, protein content and amino acid composition across all treatments. (Table A in S1 File) Control and amended feeds were all manufactured using the same process, being pelleted first and then crumbled.

Each treatment was applied to five birds in each of four pens (ie. 20 birds per diet treatment). Treatments involved the unsupplemented commercial layer diet (control group) and this ration amended with biochar (BC), bentonite (ZT) and zeolite (ZT) at 4% w/w, with adjustment to maintain the same feed value (calcium, protein, essential amino acids and metabolisable energy levels) to each group with n = 20 birds. Additives were blended into the ration by Blue Ribbon Stocks Feed, Rockhampton Qld, using a grinder, mixer and feed pelletiser. Biochar was sourced from Pacific Pyrolysis (Sydney), being woody green waste subjected to pyrolysis at 550°C. The biochar contained 76.1% C, 3.16% H, 0.29% N and 0.03% S, and effective cation exchange capacity (ECEC) of 29.7 cmol+/kg. Bentonite was sourced from JNJ Resources P/L (Brisbane, Qld) who market bentonite as a binder for pelletising feed (typically used at up to 2% w/w) and as a feed additive to bind toxins and for alleviation of urea poisoning (through its cation exchange capacity). The material has a claimed ammonia exchange capacity of 76 meq/100g DW, total composition of 67% SiO2 and 22% w/w Al_2_O_3_ (Table B in S1 File). Zeolite was sourced from Castle Mountain Zeolite (Quirindi, NSW). The material is marketed for use with poultry to increase nitrogen use efficiency and for binding of dietary toxins (http://www.cmzeolites.com.au/animals/poultry). The material has a claimed ammonia exchange capacity of 156 meq/100g DW, a total composition of 72% SiO2 and 12% w/w Al2O3, and a zeolite composition of 85% clinoptilolite and 15% mordenite. An X ray diffraction analysis suggested a composition of 17% plagioclase feldspar, 20% quartz, 5% pyroxene and 56% w/w of a zeolite likely to be clinoptilolite.

The treatment was continued for 23 weeks until the birds were 41 weeks of age. Birds were housed in a commercial layer caging system (each cage being 60×60×50 cm in height, width and depth, respectively). Pullets were randomly assigned to the cages, with five birds in each unit. Four pens with five birds each were used for each treatment, within a randomised block layout. Thus, a total of 20 birds were used for each treatment.

Water was supplied via two ‘on-demand’ nipples per cage. Feed of known weight was supplied daily, with unused material weighed. Birds were weighed individually every 15 days. Eggs were collected daily, counted and weighed.

### Microbial sampling and DNA preparation

Cloacal samples were taken using a sterile swab at 37 weeks of bird age. DNA samples from each group were selected for 16S rRNA gene amplicon generation and sequencing. Removing samples with low sequence number resulted in n = 12–15 per treatment for the microbiota analysis.

Total DNA was isolated using Bioline ISOLATE Faecal DNA Kit (#BIO-52038) according to the manufacturer’s instructions. DNA amplification was performed using Q5 DNA polymerase (New England Biolabs). Sequencing was completed on an Illumina MiSeq system (2 x 300 bp) using the dual-indexing, variable spacer, method detailed by Fadrosh *et al*. [28]. Sequencing outputs were analysed in Qiime version 1.9.1 software [29] using Qiime default parameters except for split library demultiplexing where only sequences with Phred quality threshold higher than 20 were retained for analysis (default is 3). OTUs were picked using the Uclust algorithm [30] and inspected for chimeric sequences using Pintail [31]. Taxonomy was assigned using blast against GreenGenes database [32]. Additional taxonomic assignment was done using a command line version of blastn [33] against the 16S Microbial database. The complete dataset for this experiment is publically available on the MG-RAST server under project ID 4693702.3.

The analysis was performed using data rarefied to 1850 sequences per sample removing samples with lower coverage. Statistical comparisons of the microbiota composition between the different treatment groups were performed using ANOVA on square root transformed data and Tukey Honest Significant Differences (HSD) test was performed for individual group to group comparisons in R software (https://cran.r-project.org/). Alpha diversity comparisons between birds of different treatment groups were calculated using a two-sample nonparametric t-test and up to 10^4^ Monte Carlo permutations. Beta diversity statistics was based on Adonis and up to 10^6^ permutations. Some data was visualised using Calypso (http://bioinfo.qimr.edu.au/).

## Results and Discussion

### Bird performance

There were no significant differences in bird weight between the four groups either before (*p* = 0.284) or after (*p* = 0.905) the period of diet supplementation (Fig A in S1 File). Egg production was higher in the groups with the additives, but differences between feed additive types were not statistically significant. Although there was no significant difference in number of eggs (*p* = 0.053), all three treatments had higher egg production than control. Average egg weight was significantly (*p* = 0.03) different between the treatments and higher in all 3 treatments than in control. Control group had highest feed intake (*p* = 0.001). FCR was significantly better (*p* = 0.007) in all 3 treatments, with 2.12, 2.2 and 2.17 for BC, BT and ZT respectively, compared with control that required 2.4 kg feed/kg egg (Table C in S1 File).

### Microbiota response to additives

The most highly represented phyla within the cloacal microbiota of the laying hens were Actinobacteria (41%), Firmicutes (37%), Proteobacteria (13%) and Bacteroidetes (4%), while phyla present in low abundance (<0.01%) included Fusobacteria, Tenericutes, Verrucomicrobia and TM7, with traces of Thermi, Elusimicrobia, Deferribacteres and Gemmatimonadetes.

Feed amendment with biochar, bentonite or zeolite did not result in altered microbial community richness and diversity compared to the normal unsupplemented feed group. The indices inspected included richness and evenness index (Fig B in S1 File), Shannon, Simpson, Chao1, dominance, observed species and Fisher’s alpha. The lack of influence on richness and diversity was evident at all phylogenetic levels.

**Fig 1:**
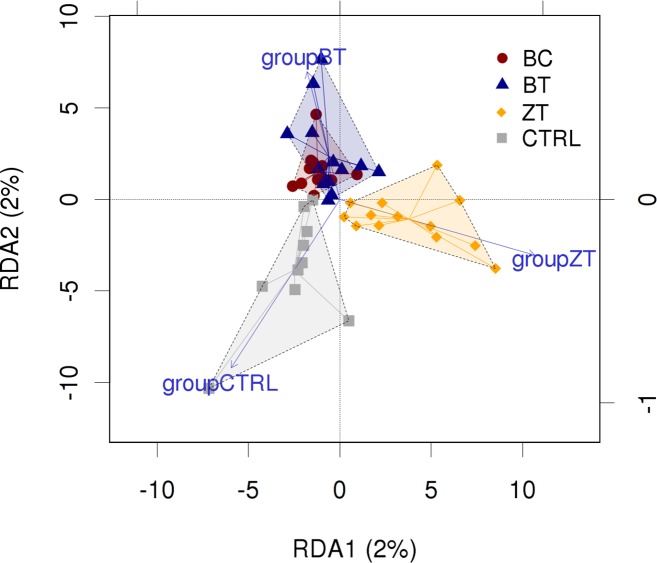
OTU level redundancy analysis (RDA) plot comparing chicken cloacal samples of birds fed control diet (CTRL) and groups with feed supplemented with biochar (BC), bentonite (BT) and zeolite (ZT). Although the ordination plot shows some separation of control and ZT groups and strong overlap of BC and BT, the first and second ordination axes represent only 2% of the variability in the data set and the separation of the groups was not statistically significant (*p* = 0.143, 1999 permutations).

There were no major community shifts caused by addition of BC, BT or ZT as shown by either unweighted (*p* = 0.2019) or weighted (*p* = 0.4817) UniFrac. A redundancy analysis (RDA) ordination plot (Fig 1) showed slight differentiation (*p* = 0.143, 1999 permutations) between the control and supplemented groups. However, individual phylotypes at different phylogenetic levels did respond differentially to BC, BT and ZT.

**Fig 2:**
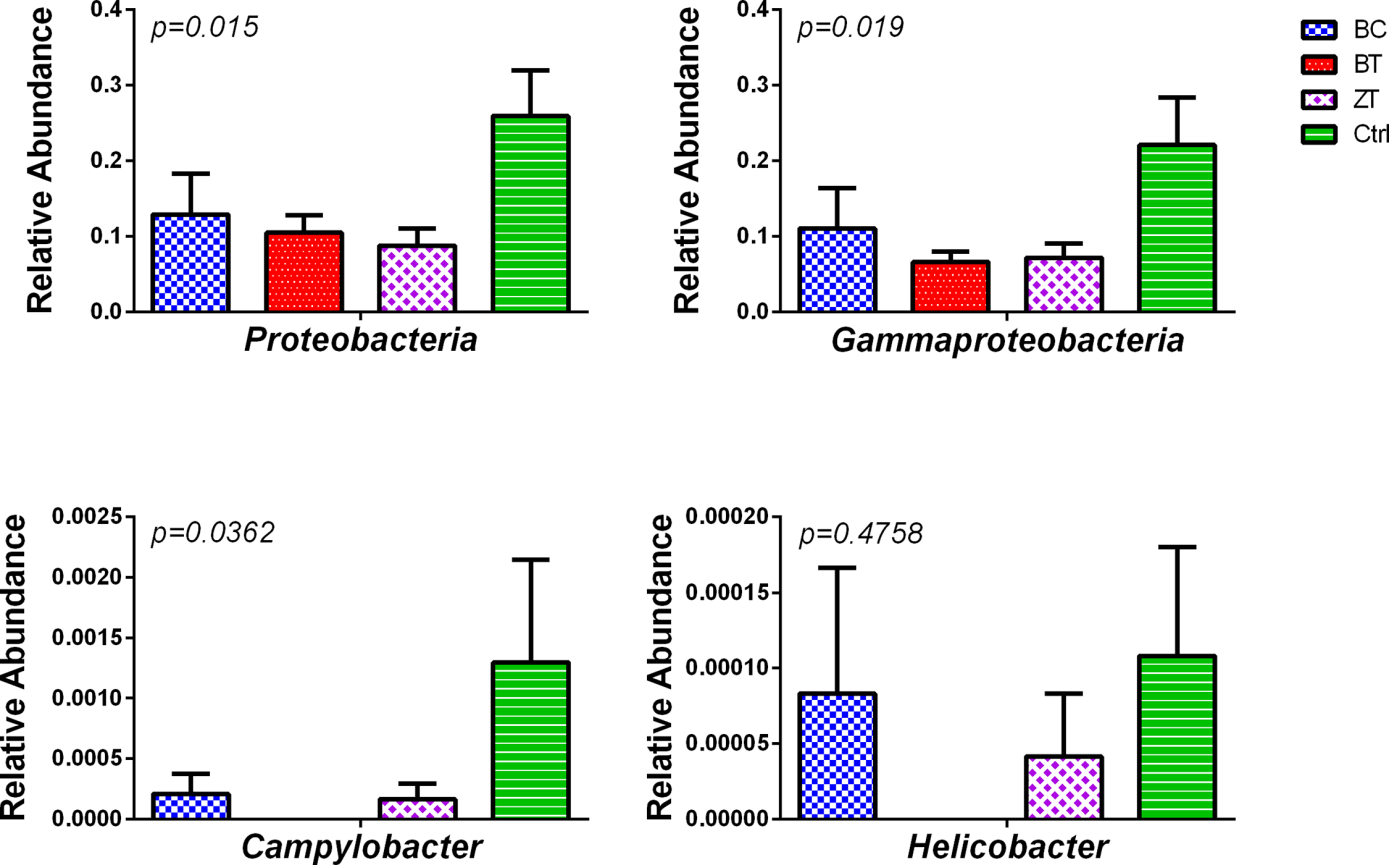
Influence of biochar (BC), bentonite (BT) and zeolite (ZT) feed supplementation on abundance of phylum Proteobacteria in chicken cloaca. Phylum Proteobacteria was significantly different between the three additives and control. The reduction of Proteobacteria in additive groups was due to significant alterations in two of its classes: Epsilonproteobacteria (*p* = 0.0179) and Gammaproteobacteria (*p* = 0.0191) (top right panel). The Epsilonproteobacteria was comprised of only two genera—*Campylobacter* and *Helicobacter* (bottom row), each represented with only one species. *Campylobacter* OTU269490 was 100% identical to *Campylobacter jejuni* subsp. *jejuni* NCTC 11168 = ATCC 700819 strain, while *Helicobacter* OTU574168, (not significantly altered, *p* = 0.4758), was identified as 99% identical *H*. *pullorum*. *Campylobacter jejuni* was reduced from mean of relative abundance 0.0013, equalling 1.3%, in control diet fed birds to mean of 0.02% in BC, completely absent in BT and down to 0.016% in ZT. The bars represent standard error for n>12.

The only phylum significantly affected by the feed additives was Proteobacteria. The prevalence of this phylum was reduced (*p* = 0.015) in all three additive groups compared to the control group (Fig 2). A Tukey HSD test showed that only ZT (*p* = 0.015) and BC (*p* = 0.0445) were statistically significantly different to the control group, while the BT group (*p* = 0.0512) was just above the 0.05 *p*-value cut-off. The reduction of Proteobacteria was due to significant alterations in abundance of the classes Epsilonproteobacteria (*p* = 0.0179) and Gammaproteobacteria (*p* = 0.0191) (Fig 2). The Epsilonproteobacteria was comprised of one order, Campylobacterales (*p* = 0.0179), which was comprised of only two OTUs (OTU269490 and OTU574168) belonging to *Campylobacter* and *Helicobacter* genera, respectively (Fig 2). *Salmonella* sp. were detected in only few birds with <5 sequences in each bird and were removed during the filtering steps.

OTU269490 (*p* = 0.0362), was identified, using blastn against the 16S Microbial database, as 100% identical to *Campylobacter jejuni* subsp. jejuni NCTC 11168 = ATCC 700819 strain across the 405nt length of the amplified OTU sequence. Although the BC, BT and ZT groups all had lower *Campylobacter* numbers compared to the control group, only the BT group was statistically significant in that reduction (Tukey HSD, *p* = 0.0244).

OTU574168 (not significantly altered, *p* = 0.4758) was identified as 99% identical to *Helicobacter pullorum*. (Fig 2). Despite *Helicobacte*r being undetected in the BT group there was no statistically significant difference (*p* = 0.4069) to the control group due to high variation in control group and not all birds having *Helicobacte*r present.

**Fig 3:**
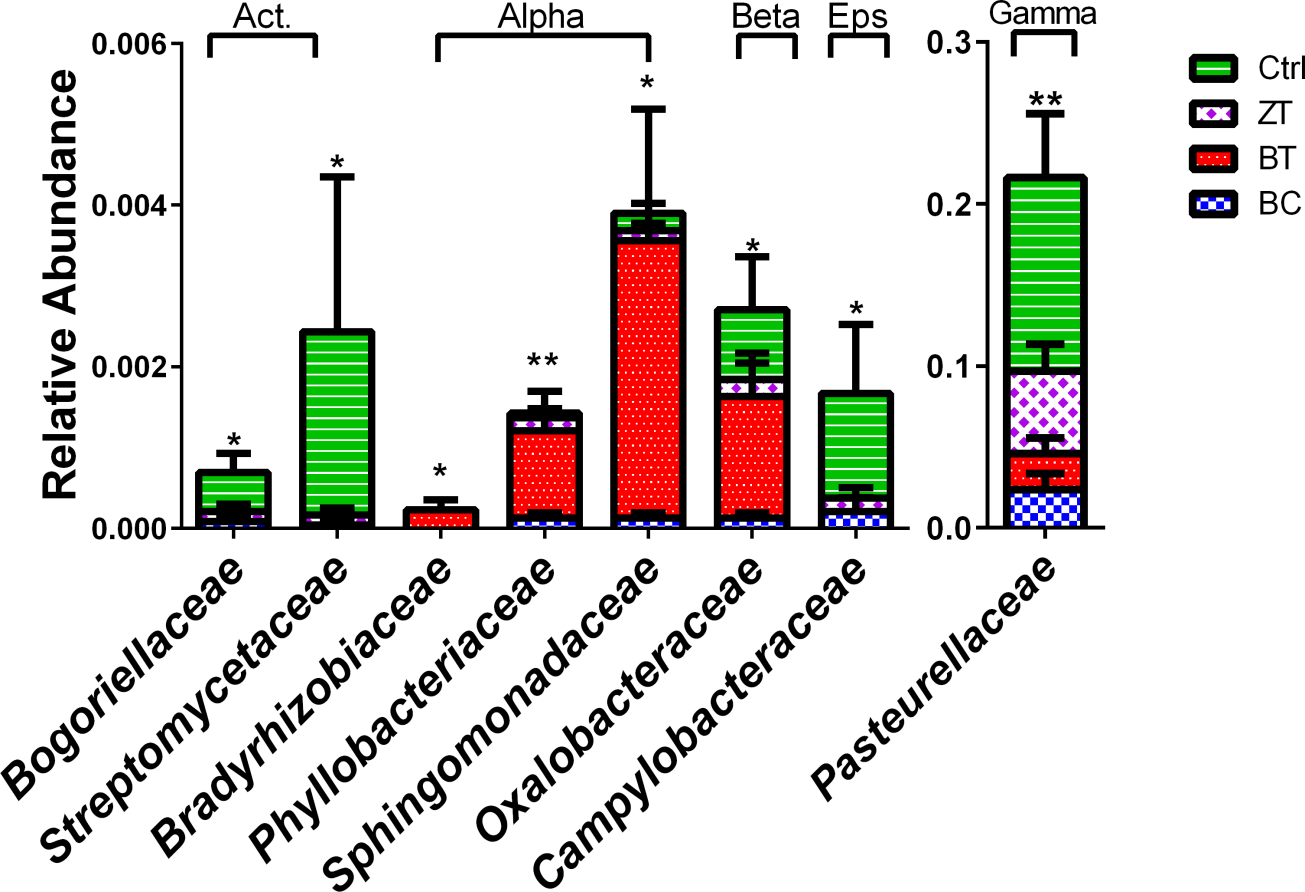
Bacterial families significantly (*p*<0.05) differed between birds fed control diet (Ctrl) and groups with feed supplemented with biochar (BC), bentonite (BT) and zeolite (ZT). The families altered were members of order Actinomycetales (marked with “Act” above the bar chart) or phylum Proteobacteria’s Alphaproteobacteria, Betaproteobacteria, Epsilonproteobacteria or Gammaproteobacteria (also marked above the bar chart). Families induced in BT Bradyrhizobiaceae, Phyllobacteriaceae, are plant-associated bacteria, Sphingomonadaceae is a candidate for bioremediation and Oxalobacteraceae are known as nitrogen fixing. The bars represent standard error for n>12.

**Fig 4:**
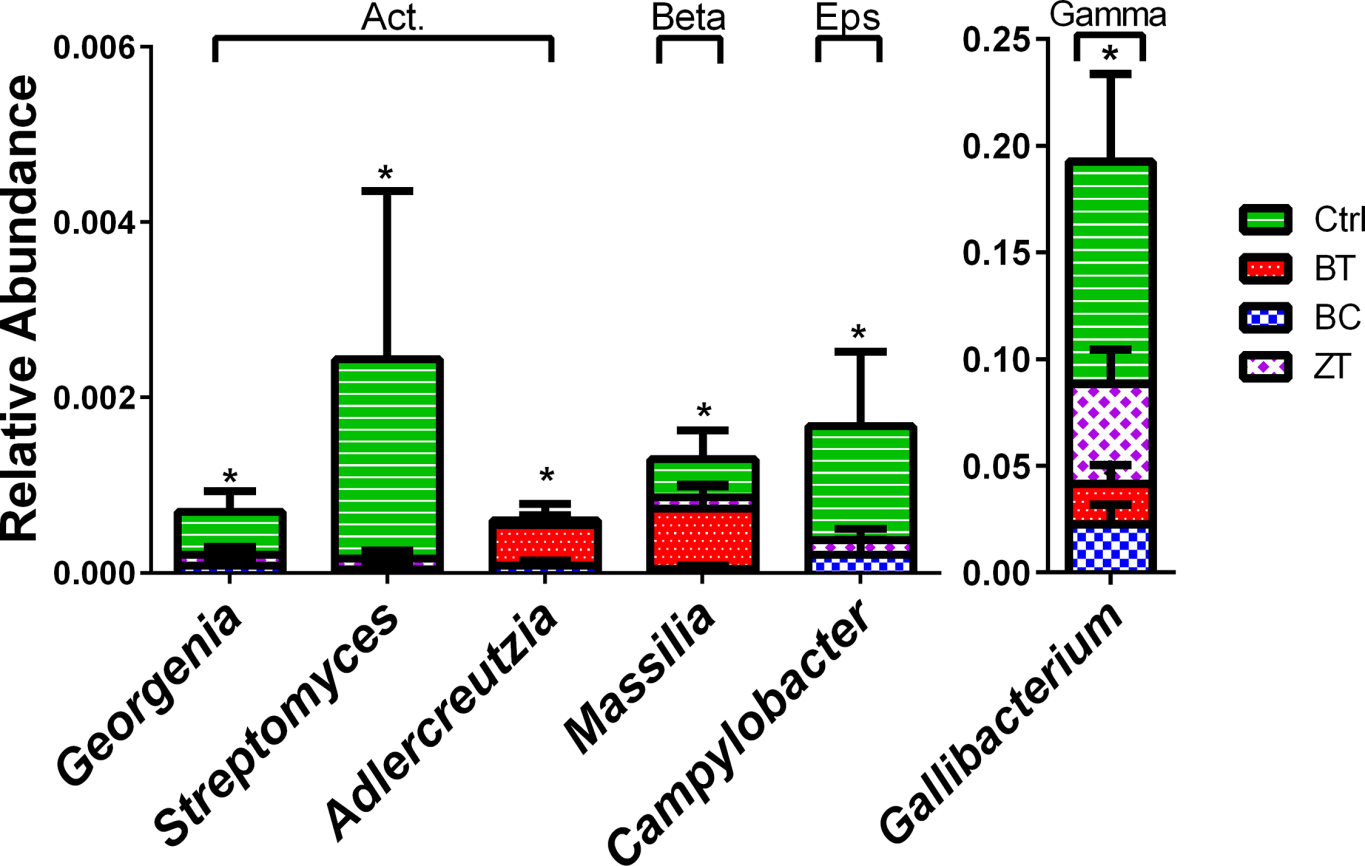
Bacterial genera significantly (*p*<0.05) differed between birds fed control diet (Ctrl) and groups with feed supplemented with biochar (BC), bentonite (BT) and zeolite (ZT). Members of order Actinomycetales are marked with “Act” above the bars chart, while other marking indicates genera belonging to Betaproteobacteria (Beta), Epsilonproteobacteria (Eps) or Gammaproteobacteria (Gamma). The bars represent standard error for n>12.

**Fig 5:**
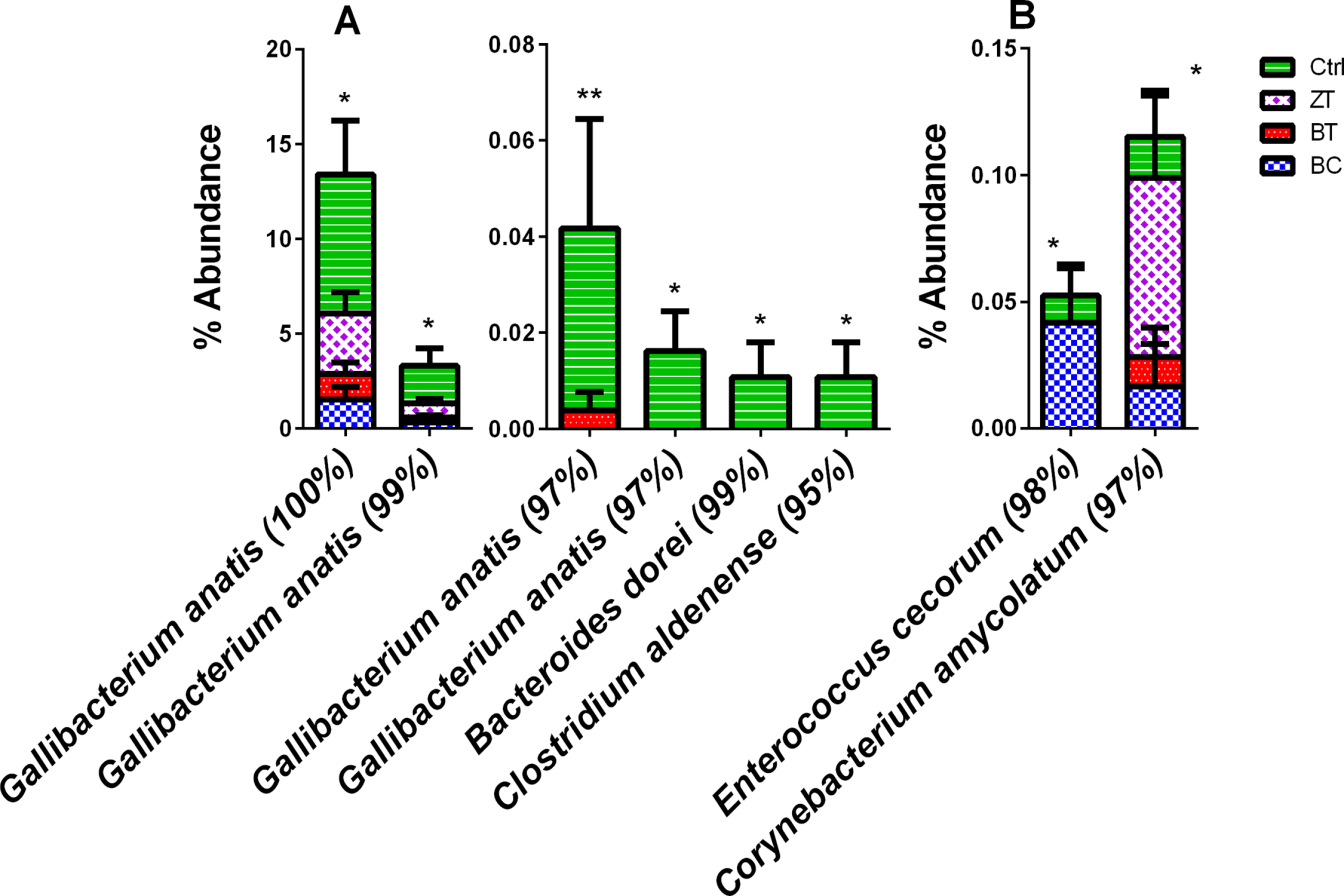
OTUs significantly (p<0.05) differed between the groups with high sequence alignment identity (>95%) with known pathogenic strains. Out of 65 OTUs significantly altered between the treatment groups (Fig C in S1 File), the majority could not be provisionally assigned to species or genus level using 97% or 95% similarity cut-off. Among the phylotypes with sequence similarity >95%, some candidates showed high sequence alignment (blast against 16S microbial database) to known pathogens. Panel A shows potentially pathogenic OTUs reduced in additives while panel B shows potentially pathogenic OTUs increased in additive groups. Y axes indicate % of abundance in each group. The bars represent standard error for n>12.

Families (Fig 3) and genera (Fig 4) significantly altered were either members of order Actinomycetales or phylum Proteobacteria. There were 65 OTUs that were differentiated in abundance between the treatment groups and control group. Blast taxonomic assignments and bar charts showing the distribution between the groups in each of the significantly altered OTUs are shown in Table D and Fig C in S1 File. Only 17 of the 65 significantly altered OTUs showed sequence similarity to entries in the 16S Microbial database with higher than 95% similarity. Out of the differentially abundant phylotypes with sequence similarity >95% there were a number of OTUs that represented known potential pathogens that were either reduced (six OTUs, Fig 5A) or increased (two OTUs, Fig 5B) by BC, BT and/or ZT.

The uses of biochar, bentonite and zeolite as feed additives potentially have multiple benefits for the chickens. Here we present the first sequencing-based, culture-independent assessment of their effects on the gut microbiota. Most of the classic antimicrobial growth promoters have a broad spectra of action. Thus they reduce richness and diversity of the intestinal microbiota, which is a risk factor for dysbiosis and other gastrointestinal complications [34]. We demonstrated that biochar, bentonite and zeolite can be used to selectively reduce the abundance of some major poultry zoonotic pathogens without reducing chicken microbiota diversity or introducing major microbiota shifts. This is highly relevant for the health of the animals and indeed to human health.

Biochar, bentonite and zeolite treatment groups all had lower levels of an OTU identified as 100% identical to *Campylobacter jejuni*. Although the biochar and zeolite groups did not meet the statistical criteria of significance due to the high degree of variation in carriage in the control group, the reduction in the bentonite group was complete and statistically significant. The Campylobacteriaceae family includes many human and animal pathogenic species; the most significant *C*. *jejuni*, is recognized as a leading cause of foodborne infections. The European Food Safety Authority (EFSA) has estimated that within the European Union there are 9 million cases of *Campylobacter* food poisoning annually, with related cost of EUR 2.4 billion per year, 20–30% of which cases are attributed to contaminated chicken meat [35].

Shifts and dysbiosis in intestinal microbiota are reported as the first steps in successful colonization by *C*. *jejuni* [36, 37] and possible disease onset. The bacterium is a common member of the intestinal microbiota in wildlife and agricultural animals, where it usually exists asymptomatically. These animals thus represent a reservoir of this major zoonotic human disease.

Additionally, we detected the ability of bentonite to reduce a *Helicobacter pullorum*–related OTU. This species, which was completely absent from the gut of all birds on diet supplemented with bentonite, aligned with 99% similarity to *H*. *pullorum*. This enterohepatic helicobacter species is found in the gut of healthy chickens as well as in liver and intestine of hens with vibrionic-like liver lesions [38] and in human chronic liver disease [39]. It is also associated with human gastroenteritis, inflammatory bowel disease and Chron’s disease [40–42]. The ability to reduce both *Helicobacter* and *Campylobacter*, if reproducible under varied feeding and environmental conditions, would be a compelling reason to include bentonite in poultry feed formulations.

Biochar and zeolite also affected the carriage of these potentially pathogenic species, although not as significantly as bentonite. Zeolite was found to significantly reduce the levels of the phylum Proteobacteria. High numbers of Proteobacteria are considered a sign of bad intestinal health and are associated with gastrointestinal health conditions such as chronic dysbiosis [43] and inflammatory bowel disease [44].

All three additives reduced the abundance of four OTUs identified with high sequence identity (>97%) to *Gallibacterium anatis*, another major poultry pathogen [45]. *G*. *anatis* has been associated with a range of pathological lesions mostly in breeding chicken, including peritonitis, oophoritis, septicemia, follicle degeneration, salpingitis and respiratory tract infections [46]. Together with *Pasteurella multocida*, *G*. *anatis* causes fowl cholera-like clinical manifestations and lesions. Although *P*. *multocida* was not identified at the species level, the family Pasteurellaceae was abundant (higher than 20% in control, Fig 3) and was significantly reduced in all three additive groups, most significantly with bentonite (Fig 3).

*Bacteroides dorei* (99% identity) and *Clostridium aldenense* (95% identity) were present in control birds only, as low abundance taxa, and were absent from microbiota in all three additive groups. *Clostridium aldenense* is another pathogen involved in multiple clinical presentations including bacteremia [47, 48]. If further investigated and confirmed, the ability of additives to remove *B*. *dorei* would be of interest for human health. For example, *B*. *dorei* was found to dominate gut microbiome prior to onset of autoimmunity in children at high risk for type 1 diabetes [49, 50].

*Enterococcus cecorum* and *Corynebacterium amycolatum* are potential pathogens that were increased in some additive groups. *E*. *cecorum* was increased in the biochar group and *C*. *amycolatum* in the zeolite group. *E*. *cecorum* is an animal pathogen [51] as well as opportunistic human pathogen [52–54]. In chickens it is a major cause of outbreaks of arthritis and osteomyelitis worldwide [55]. *C*. *amycolatum* has been occasionally associated with infective endocarditis [56]. There were no affected OTUs similar to known pathogens with >95% sequence similarity that were significantly increased in bentonite.

## Conclusions

The data presented here indicate that zeolite, biochar, and in particular bentonite may be viable alternatives to AGPs in poultry and other agricultural industries that could be further developed to help control pathogen load in domestic animals without significantly changing the overall complexity of gut microbiota. Of special interest is the association of bentonite with decreased *Campylobacter* and *Helicobacter* genera. This is especially promising since bentonite has been used and proven as safe through occasional use in poultry feeds [57, 58] for purposes other than pathogen control, mostly to improve feed manufacture [10] and for reduction of the feed passage rate through the chicken gut [15].

## Supporting Information

S1 File (PDF)
