# Supplementary material for: Biochar, Bentonite and Zeolite Supplemented Feeding of Layer Chickens Alters Intestinal Microbiota and Reduces Campylobacter Load
Source: PLoS One. 2016 Apr 26;11(4):e0154061. doi: 10.1371/journal.pone.0154061 (PMC4845986; doi:10.1371/journal.pone.0154061)
Supplement: S1 File — (PDF) [file pone.0154061.s001.pdf]

Biochar, bentonite and zeolite supplemented feeding of layer chickens alters intestinal microbiota and reduces *Campylobacter* load

Tanka P. Prasai[1], Kerry B. Walsh[1], Surya P. Bhattarai[1], David J. Midmore[1], Thi T. H. Van[2], Robert J. Moore[2,3], Dragana Stanley[1*]

Supplementary Material

[1]Central Queensland University, Institute for Future Farming Systems, Rockhampton, Queensland, Australia.

[2]RMIT University, School of Applied Sciences and Health Innovations Research Institute (HIRI), Bundoora, Victoria, Australia.

[3]Department of Microbiology, Monash University, Clayton, Victoria Australia.

Corresponding author
Dragana Stanley
Central Queensland University
Bruce Highway
School of Medical and Applied Sciences
Building 6 Room 2.33
Rockhampton QLD 4702
Email: D.Stanley@cqu.edu.au
Phone: +61 7 4923 2079

Fig. A

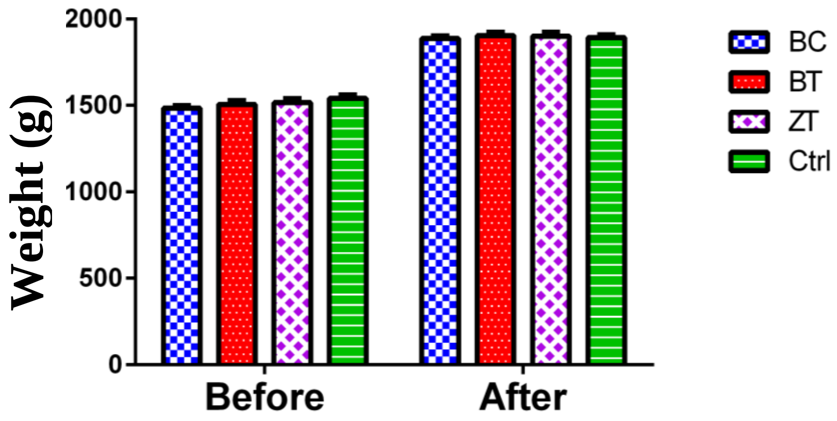

Fig. A: Bird weights were not different between the treatment groups before (*p*=0.0284) or after (*p*=0.905) 23 weeks of the diet treatment. The bars represent standard error.

Fig. B

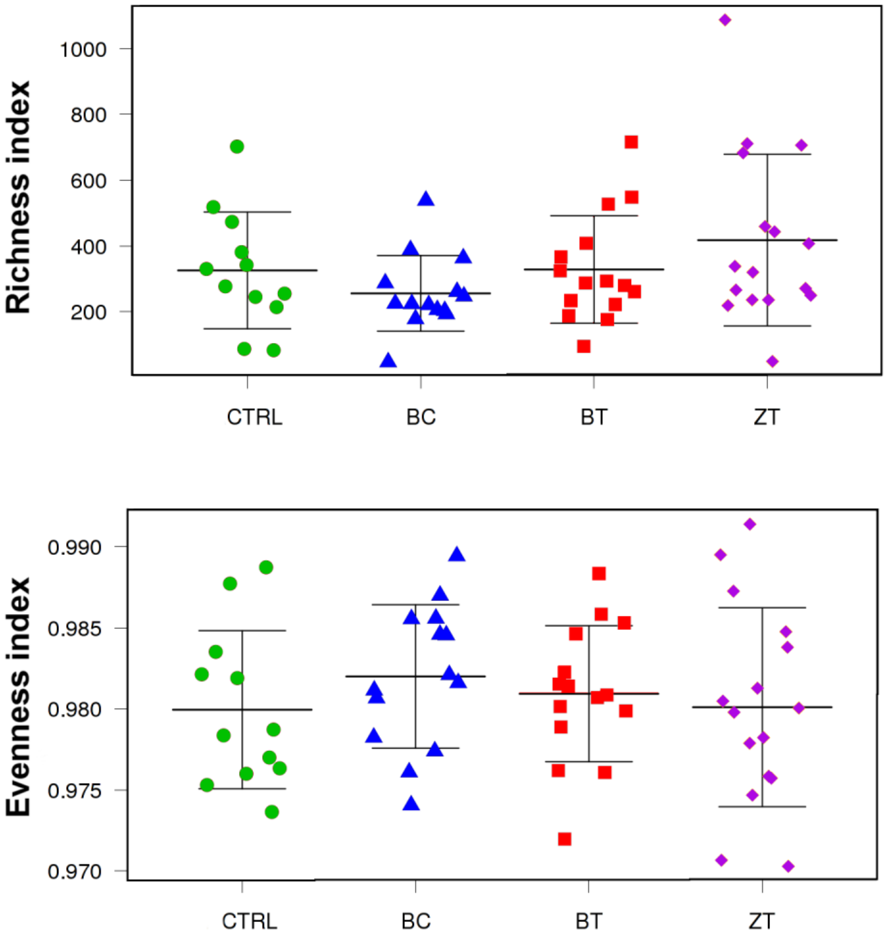

Fig. B: Addition of biochar (BC), bentonite (BT) and zeolite (ZT) to feed did not influence richness and diversity of the intestinal microbiota compared to un-supplemented control (CTRL). Top graph represents richness and bottom graph evenness index. Neither index were significantly (p<0.05) altered with additives.

Fig. C

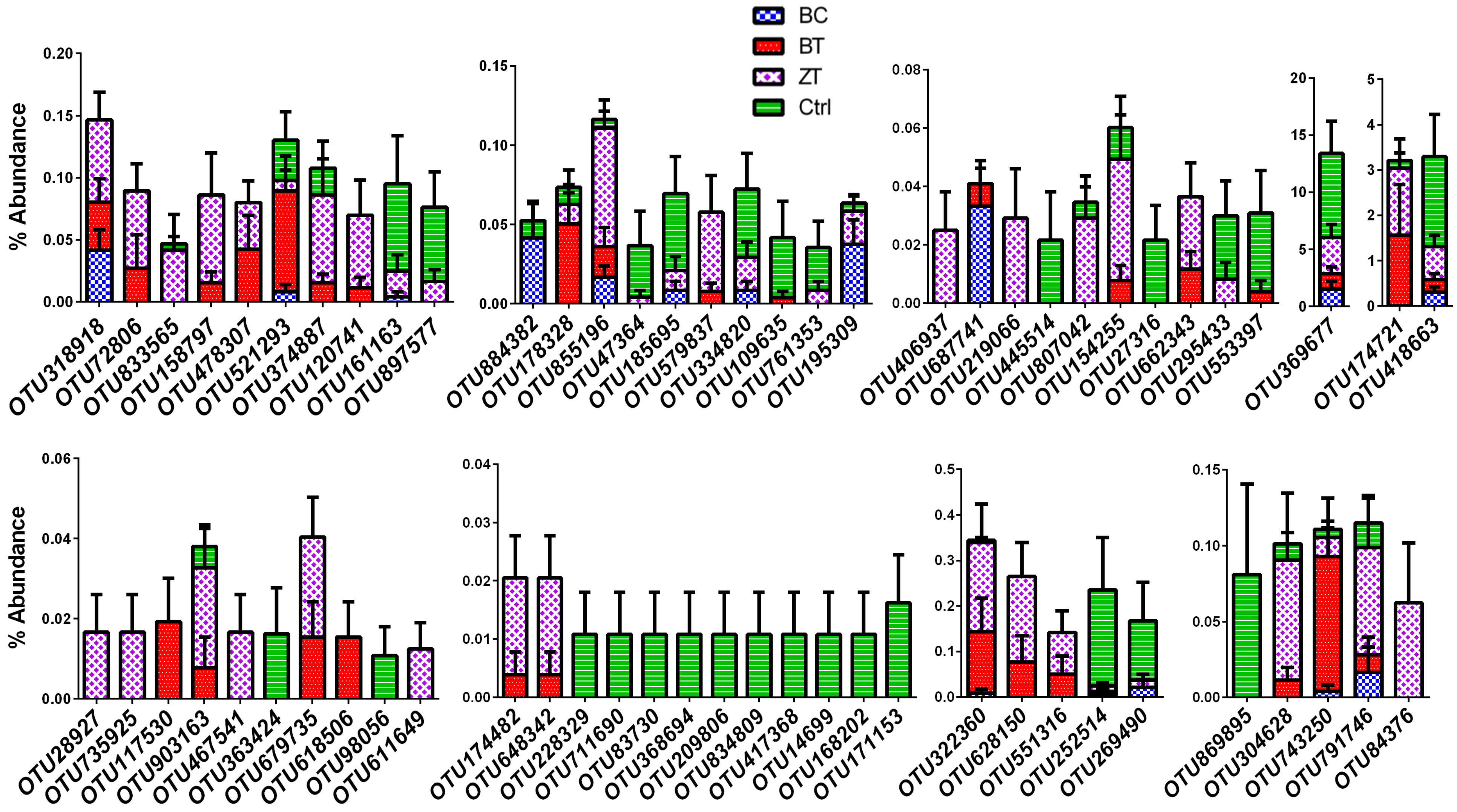

Fig. C: **OTUs differentially abundant (Qiime based ANOVA) between the diet treatment groups.** ***P*****-values and closets blast aligned strain from 16S Microbial database are given in the Table D.** The bars represent standard error for n>12.

Table A: Composition of the layer diets (%w/w)

	Control	Biochar 4 %	Zeolite 4 %	Bentonite 4 %
Dry matter (%)	90.37	90.70	90.63	90.67
Crude fat (%)	4.09	4.29	4.25	4.19
Crude ash (%)	13.06	16.68	16.34	16.83
Crude fibre (%)	3.17	3.02	2.68	2.38
Metabolisable energy (MJ/kg)	11.75	11.40	11.53	11.50
Crude protein (%)	17.91	17.74	17.81	17.52
Calcium (%)	4.23	4.27	4.25	4.30
Phosphorus available (%)	0.47	0.45	0.46	0.45
Lysine (%)	0.91	0.94	0.99	1.00
Methionine (%)	0.45	0.43	0.45	0.45
Methionine plus cysteine (%)	0.68	0.66	0.69	0.69

Poultry premix provided per kilogram of diet; Vitamin A, 15000 IU; Vitamin D3, 5,000 IU;
Vitamin E, 50 mg; Vitamin K3, 10 mg; thiamine, 4 mg; riboflavin 8 mg; pyridoxine, 5 mg;
Vitamin B12, 0.025; niacin, 50 mg; Ca-pantothenate, 20 mg; folic acid, 2 mg; biotin, 0.25 mg;
ascorbic acid, 75 mg; choline, 175 mg; Mg, 35 mg; Mn, 56 mg; Zn, 140 mg; Fe, 56 mg;
Cu, 10.5 mg; I, 1 mg; Co, 0.28 mg; Se, 0.28 mg; Mo, 0.7 mg

Table B: Sodium Bentonite Typical Chemical (oxide) analyses (dry basis)

SiO2	66.80%	K2O	0.49%
A12O3	22.30%	TiO2	0.48%
Fe2O3	3.26%	Mn3O4	0.01%
CaO	1.48%	SO3	0.08%
MgO	2.59%	P2O5	0.05%
Na2O	2.48%	BaO	0.08%

Cation Exchange Capacity	
Minimum	70meq/100mg
Average	76meq/100mg
pH	Average 10.2
**Physical**	**Properties**
Grit Content	<3.0%
Total Moisture	Maximum 10.5%
Bulk Density	1.2kg/1

Exchangeable	Cations
Sodium	65.60%
Calcium	20.20%
Magnesium	12.40%
Potassium	1.80%
**Sizing**	**Analysis**
-2 mm	15%
-1.0 + 0.5 mm	32%
-0.5 mm	53%

Table C: Egg productivity and feed conversion ratio (FCR) across the 23 weeks of amended diet

Treatment	Egg production (% hens/day)	Average egg weight (g)	Egg mass yield (g/hen/day)	Feed intake (g/hen/day)	FCR (g feed/ g egg)	Peak egg yield (% hens/day)
Biochar 4 %	84.8	61.0	51.7	109.7	2.12	89.3
Bentonite 4 %	85.2	61.9	52.7	116.3	2.20	87.1
Zeolite 4 %	85.8	62.3	53.5	116.2	2.17	88.6
Control	83.6	59.2	49.4	118.6	2.40	87.3
SEM	0.492	0.41	0.389	0.238	0.014	0.938
P values	0.053	0.03	0.001	0.001	0.007	0.366

* P values are based on Analysis of variance (ANOVA) for 20 birds in 4 pens for each treatment

Egg mass yield is calculated as egg production x average egg weight

FCR is calculated as feed intake / egg mass yield

Peak egg yield is the highest recorded

Table D: OTUs differentially abundant (Qiime based ANOVA, shown in S3 Fig.) between the diet treatment groups. The best blastn aligned strain and % ID against 16S Microbial database are given followed with Qiime derived blastn GreenGenes taxonomy assignment.

OTU ID	p-Value	Blastn against 16S Microbial database	%ID	Qiime GreenGenes assignment
**OTU369677**	0.028	*Gallibacterium anatis* UMN179 strain UMN179	100	Proteobacteria/Gammaproteobacteria/Pasteurellales/Pasteurellaceae/*Gallibacterium/Gallibacterium anatis*
**OTU174721**	0.050	*Clostridium fimetarium* strain Z-2189	94	Firmicutes/Clostridia/Clostridiales/Lachnospiraceae
**OTU418663**	0.005	*Gallibacterium anatis* strain F 149	99	Proteobacteria/Gammaproteobacteria/Pasteurellales/Pasteurellaceae/*Gallibacterium/Gallibacterium anatis*
**OTU322360**	0.037	*Ruminococcus lactaris* strain ATCC 29176	94	Firmicutes/Clostridia/Clostridiales/Lachnospiraceae
**OTU252514**	0.025	*Rothia nasimurium* strain CCUG 35957	91	Actinobacteria/Actinobacteria/Actinomycetales/Micrococcaceae/*Rothia*
**OTU269490**	0.011	*Campylobacter jejuni* subsp. *jejuni* NCTC 11168 = ATCC 700819	100	Proteobacteria/Epsilonproteobacteria/Campylobacterales/Campylobacteraceae/*Campylobacter*
**OTU628150**	0.035	*Clostridium populeti* strain 743A	94	Firmicutes/Clostridia/Clostridiales/Lachnospiraceae
**OTU551316**	0.014	*Clostridium algidixylanolyticum* strain SPL73	95	Firmicutes/Clostridia/Clostridiales/Lachnospiraceae/*Clostridium*
**OTU869895**	0.028	*Clostridium orbiscindens* DSM 6740 strain 265	96	Firmicutes/Clostridia/Clostridiales/Ruminococcaceae/*Clostridium*
**OTU304628**	0.021	*Lactobacillus crispatus* ST1 strain ST1	95	Firmicutes/Bacilli/Lactobacillales/Lactobacillaceae/Lactobacillus/*Lactobacillus helveticus*
**OTU743250**	0.037	*Aminobacter niigataensis* strain DSM7050	97	Proteobacteria/Alphaproteobacteria/Rhizobiales/Phyllobacteriaceae
**OTU791746**	0.023	*Corynebacterium amycolatum* strain CIP 103452	97	Actinobacteria/Actinobacteria/Actinomycetales/Corynebacteriaceae/*Corynebacterium*
**OTU84376**	0.009	*Phascolarctobacterium faecium* strain ACM 3679	90	Firmicutes/Clostridia/Clostridiales/Veillonellaceae/*Phascolarctobacterium*
**OTU72806**	0.019	*Actinomyces nasicola* strain R2014	90	Actinobacteria/Actinobacteria/Actinomycetales/Actinomycetaceae/*Actinomyces/Actinomyces marimammalium*
**OTU833565**	0.037	*Peptoniphilus ivorii* strain DSM 10022	92	Firmicutes/Clostridia/Clostridiales/ClostridialesFamilyXI.IncertaeSedis/*Peptoniphilus*
**OTU158797**	0.032	*Ruminococcus lactaris* strain ATCC 29176	95	Firmicutes/Clostridia/Clostridiales/Lachnospiraceae
**OTU478307**	0.037	*Clostridium fimetarium* strain Z-2189	91	Firmicutes/Clostridia/Clostridiales/Lachnospiraceae
**OTU521293**	0.007	*Ralstonia syzygii* strain ATCC 49543	97	Proteobacteria/Betaproteobacteria/Burkholderiales/Oxalobacteraceae
**OTU374887**	0.022	*Actinomyces nasicola* strain R2014	90	Actinobacteria/Actinobacteria/Actinomycetales/Actinomycetaceae
**OTU120741**	0.029	*Atopobium parvulum* DSM 20469 strain DSM 20469	93	Actinobacteria/Actinobacteria/Coriobacteriales/Coriobacteriaceae
**OTU161163**	0.024	*Gallibacterium anatis* UMN179 strain UMN179	89	Proteobacteria/Gammaproteobacteria/Pasteurellales/Pasteurellaceae/*Gallibacterium/Gallibacterium anatis*
**OTU897577**	0.042	*Actinomyces nasicola* strain R2014	87	Actinobacteria/Actinobacteria/Actinomycetales/Actinomycetaceae

OTU ID	p-Value	Blastn against 16S Microbial database	%ID	Qiime GreenGenes assignment
**OTU318918**	0.040	*Actinomyces europaeus* strain CCUG 32789A	93	Actinobacteria/Actinobacteria/Actinomycetales/Actinomycetaceae/*Actinomyces/Actinomyces europaeus*
**OTU884382**	0.029	*Enterococcus cecorum* strain ATCC 43198	98	Firmicutes/Bacilli/Lactobacillales/Enterococcaceae/*Enterococcus/Enterococcus cecorum*
**OTU178328**	0.046	*Naxibacter varians* strain : CCUG 35299	99	Proteobacteria/Betaproteobacteria/Burkholderiales/Oxalobacteraceae/*Massilia/Massilia timonae*
**OTU855196**	0.043	*Actinomyces europaeus* strain CCUG 32789A	93	Actinobacteria/Actinobacteria/Actinomycetales/Actinomycetaceae/*Actinomyces/Actinomyces europaeus*
**OTU47364**	0.039	*Varibaculum cambriense* strain CCUG 44998	92	Actinobacteria/Actinobacteria/Actinomycetales/Actinomycetaceae/*Varibaculum/Varibaculum cambriense*
**OTU185695**	0.037	*Actinomyces nasicola* strain R2014	91	Actinobacteria/Actinobacteria/Actinomycetales/Bogoriellaceae/*Georgenia*
**OTU579837**	0.037	*Ruminococcus lactaris* strain ATCC 29176	96	Firmicutes/Clostridia/Clostridiales/Lachnospiraceae
**OTU334820**	0.029	*Helcococcus sueciensis* strain CCUG 47334	87	Firmicutes/Clostridia/Clostridiales/ClostridialesFamilyXI.IncertaeSedis/*Helcococcus*
**OTU109635**	0.001	*Gallibacterium anatis* UMN179 strain UMN179	97	Proteobacteria/Gammaproteobacteria/Pasteurellales/Pasteurellaceae/*Gallibacterium/Gallibacterium genomo sp.1*
**OTU761353**	0.036	*Actinomyces canis* strain CCUG 41706	93	Actinobacteria/Actinobacteria/Actinomycetales/Actinomycetaceae/*Actinomyces*
**OTU195309**	0.035	*Actinomyces europaeus* strain CCUG 32789A	93	Actinobacteria/Actinobacteria/Actinomycetales/Actinomycetaceae/*Actinomyces/Actinomyces europaeus*
**OTU406937**	0.026	*Actinomyces europaeus* strain CCUG 32789A	93	Actinobacteria/Actinobacteria/Actinomycetales/Actinomycetaceae/*Actinomyces/Actinomyces europaeus*
**OTU687741**	0.004	*Barnesiella intestinihominis* YIT 11860 strain YIT 11860	88	Bacteroidetes/Bacteroidia/Bacteroidales
**OTU219066**	0.033	*Ruminococcus lactaris* strain ATCC 29176	97	Firmicutes/Clostridia/Clostridiales/Lachnospiraceae
**OTU445514**	0.005	*Clostridium xylanolyticum* strain DSM 6555	92	Firmicutes/Clostridia/Clostridiales/Lachnospiraceae
**OTU807042**	0.049	*Parasporobacterium paucivorans* strain SYR1	90	Firmicutes/Clostridia/Clostridiales/Lachnospiraceae
**OTU154255**	0.049	*Ruminococcus torques* strain VPI B2-51	86	Firmicutes/Clostridia/Clostridiales/Lachnospiraceae/*Ruminococcus*
**OTU27316**	0.049	*Actinomyces canis* strain CCUG 41706	90	Actinobacteria/Actinobacteria/Actinomycetales/Actinomycetaceae
**OTU662343**	0.045	*Actinomyces europaeus* strain CCUG 32789A	94	Actinobacteria/Actinobacteria/Actinomycetales/Actinomycetaceae/Actinomyces/Actinomyces europaeus
**OTU295433**	0.046	Rothia nasimurium strain CCUG 35957	92	Actinobacteria/Actinobacteria/Actinomycetales/Micrococcaceae/*Rothia*
**OTU553397**	0.044	*Enterococcus avium* strain E6844	88	Actinobacteria/Actinobacteria/Actinomycetales/Micrococcaceae/*Rothia*
**OTU28927**	0.045	*Barnesiella intestinihominis* YIT 11860 strain YIT 11860	78	Bacteroidetes/Bacteroidia/Bacteroidales
**OTU735925**	0.044	*Paraprevotella clara* YIT 11840 strain YIT 11840	87	Bacteroidetes/Bacteroidia/Bacteroidales/Prevotellaceae/*Prevotella*
**OTU117530**	0.042	*Clostridium sartagoforme* strain DSM 1292	94	Firmicutes/Clostridia/Clostridiales/Clostridiaceae/Clostridium/*Clostridium* perfringens
**OTU903163**	0.042	*Peptoniphilus ivorii* strain DSM 10022	88	Firmicutes/Clostridia/Clostridiales/ClostridialesFamilyXI.IncertaeSedis/*Peptoniphilus*
**OTU467541**	0.019	*Clostridium celerecrescens* strain DSM 5628	96	Firmicutes/Clostridia/Clostridiales/Lachnospiraceae

OTU ID	p-Value	Blastn against 16S Microbial database	%ID	Qiime GreenGenes assignment
**OTU363424**	0.033	*Clostridium methylpentosum* DSM 5476 strain R2	90	Firmicutes/Clostridia/Clostridiales/Ruminococcaceae/*Clostridium*
**OTU679735**	0.037	*Faecalibacterium prausnitzii* strain ATCC 27768	90	Firmicutes/Clostridia/Clostridiales/Ruminococcaceae/*Faecalibacterium*
**OTU618506**	0.037	*Balneimonas flocculans* strain TFB	99	Proteobacteria/Alphaproteobacteria/Rhizobiales/*Bradyrhizobiaceae*
**OTU98056**	0.039	*Actinomyces marimammalium* strain CCUG 41710	88	Actinobacteria/Actinobacteria/Actinomycetales/Actinomycetaceae/*Actinomyces*
**OTU611649**	0.003	*Actinomyces europaeus* strain CCUG 32789A	95	Actinobacteria/Actinobacteria/Actinomycetales/Actinomycetaceae/*Actinomyces/Actinomyces europaeus*
**OTU228329**	0.026	*Rothia terrae* strain L-143	93	Actinobacteria/Actinobacteria/Actinomycetales/Micrococcaceae/*Kocuria*
**OTU174482**	0.009	*Galbibacter mesophilus* strain Mok-17	84	Bacteroidetes/Bacteroidia/Bacteroidales
**OTU711690**	0.013	*Bacteroides dorei* DSM 17855 strain JCM 13471	99	Bacteroidetes/Bacteroidia/Bacteroidales/Bacteroidaceae/*Bacteroides/Bacteroides dorei*
**OTU83730**	0.037	*Clostridium saccharogumia* strain SDG-Mt85-3Db	86	Firmicutes/Clostridia/Clostridiales/Clostridiaceae/*Clostridium*
**OTU368694**	0.040	*Actinomyces cardiffensis* strain CCUG 44997	85	Firmicutes/Clostridia/Clostridiales/ClostridialesFamilyXI.IncertaeSedis/*Peptoniphilus*
**OTU209806**	0.032	*Clostridium aldenense* strain RMA 9741	96	Firmicutes/Clostridia/Clostridiales/Lachnospiraceae
**OTU648342**	0.020	*Clostridium sporosphaeroides* strain DSM 1294	91	Firmicutes/Clostridia/Clostridiales/Ruminococcaceae
**OTU834809**	0.014	*Faecalibacterium prausnitzii* strain ATCC 27768	94	Firmicutes/Clostridia/Clostridiales/Ruminococcaceae/*Faecalibacterium*
**OTU417368**	0.037	*Clostridium methylpentosum* DSM 5476 strain R2	87	Firmicutes/Clostridia/Clostridiales/Ruminococcaceae/*Ruminococcus*
**OTU14699**	0.004	*Serratia marcescens* WW4 strain WW4	87	Proteobacteria/Gammaproteobacteria/Enterobacteriales/*Enterobacteriaceae*
**OTU168202**	0.008	*Corynebacterium xerosis* strain ATCC 373	88	Proteobacteria/Gammaproteobacteria/Pasteurellales/Pasteurellaceae/*Gallibacterium/Gallibacterium anatis*
**OTU171153**	0.036	*Gallibacterium anatis* UMN179 strain UMN179	97	Proteobacteria/Gammaproteobacteria/Pasteurellales/Pasteurellaceae/*Gallibacterium/Gallibacterium anatis*
